# Innovation in diagnostics: addressing gaps in low- and middle-income countries

**DOI:** 10.2471/BLT.22.288313

**Published:** 2022-08-01

**Authors:** Claudia Chamas, Mady Malheiros Barbeitas, Marilena Correa, Koichi Kameda, Ana Claudia Dias de Oliveira, Luiz Villarinho

**Affiliations:** a Oswaldo Cruz Foundation and National Institute of Science and Technology for Innovation on Diseases of Neglected Populations, Avenida Brasil, 4036, Sala 814, Rio de Janeiro RJ Brazil 21040-36, Brazil.; b École des Hautes Études en Sciences Sociales (EHESS, Cermes3), Villejuif, France.; c Social Medicine Institute, University of the State of Rio de Janeiro, Rio de Janeiro, Brazil.; d Centre Population et Développement, Institut de Recherche pour le Développement (CEPED, IRD), Paris, France.; e Innovation Department, University of the State of Rio de Janeiro, Rio de Janeiro, Brazil.; Correspondence to Claudia Chamas (email: claudia.chamas@fiocruz.br).

In vitro diagnostic tests are essential tools in the fight against the coronavirus disease 2019 (COVID-19) pandemic and other infectious disease outbreaks.^1^ However, access to effective and low-cost tests in many low- and middle-income countries is suboptimal. Improving such access depends on factors at country level such as health priorities, public procurement, research capacity, local innovation and local production. Diagnostics research and development in low- and middle-income countries is one of the most neglected topics in global health.^2^

Technology sharing models are critical to promoting technological catch-up based on public interest.^3^ A good example is the World Health Organization’s (WHO) COVID-19 Technology Access Pool, an initiative that aims to expand equitable access to COVID-19 health products, providing a global platform for developers to share knowledge, intellectual property and data. In November 2021, the technology access pool and the Medicines Patent Pool announced a licence with the Spanish National Research Council for a COVID-19 serological antibody technology. This global agreement is transparent, royalty-free and non-exclusive; it covers all the necessary patents, biological material and know-how for production on a global scale.^4^

Some countries have demonstrated technological dynamism, that is, an ability to generate and incorporate innovations, through the development and production of tests. For example, in India, the National Institute of Virology, part of the Indian Council of Medical Research, developed the COVID KAVACH ELISA kit. The technology allows the detection of immunoglobulin G antibodies in patients who present with symptoms consistent with severe acute respiratory syndrome coronavirus 2 (SARS-CoV-2) infection. A technology transfer agreement was signed with the company Zydus Lifesciences Ltd (Ahmedabad, India), which supplied the product free of cost to the Indian Council of Medical Research.^5^

Fiocruz, a public biomedical research institution based in Brazil, has recently developed two reverse transcriptase polymerase chain reaction (RT–PCR) tests: one that differentiates between influenza viruses A and B and SARS-CoV-2, and a second one that allows the detection and screening of variants of SARS-CoV-2. These technologies add to Fiocruz’s efforts to locally produce tests for SARS-CoV-2. In early 2022, Fiocruz had already supplied more than 20 million RT–PCR tests and 45 million rapid antigen tests to the country’s unified health system. Additionally, the Fiocruz COVID-19 Biobank was created to supply biological materials enabling the development of new tests, vaccines and other inputs, improving self-reliance and building resilience in the national health system.^6^^,^^7^

The Indian and Brazilian initiatives are examples of efforts to reduce the dependence on international supply chains. Moreover, these investments also contribute to strengthening national laboratories and preparing them to join international networks in new public health challenges.

Nonetheless, some challenges remain unaddressed. The poorer countries experience underinvestment in research and development, limited cooperation opportunities, and difficulties accessing data, biological materials and technology transfer. Another recurrent point is the drain of researchers to high-income countries. Such factors hinder a more regular and vigorous involvement of low- and middle-income countries in development of diagnostics and its global market.

The pandemic has taught us that without autonomy in technological production, the most vulnerable will have to wait for vaccine doses and diagnostic tests, and pay more for them. Low- and middle-income countries should be co-participants, not only recipients of technological solutions.^8^^,^^9^ Fair and equitable access will be promoted with the inclusion in international and global initiatives of local development capacity in those countries that are now poorly represented. 

The participation of these countries in technological and industrial cooperation for diagnostics has been a priority issue in WHO debates for at least a decade.^10^ Such participation would enable reduction of the dependence of these countries on importation of inputs, tests, equipment and devices associated with tests and would contribute to the development of high-quality, cost-effective tests adapted to local needs.^1^ In addition, the world’s health security depends on countries’ capacities – including diagnostic capacity – to deal with health crises. Thus, technological capabilities in diagnostics in low- and middle-income countries should be a global priority, demanding global collective action within and among countries.^11^

To address the gaps in diagnostics and other health tools – both in terms of new developments and access frameworks – implementing structural changes in the innovation system will be necessary. The sharing of intellectual property and know-how should be coupled with finance and network coordination, human resources training, public policies for innovation and capacity strengthening, aligning public health priorities with mobilization of State capacities and public–private partnerships. 
